# Operative and Oncological Outcomes of Vascular Resection and Reconstruction for Perihilar Cholangiocarcinoma

**DOI:** 10.1245/s10434-025-18137-4

**Published:** 2025-09-13

**Authors:** Edoardo Poletto, Pim B. Olthof, Frederik J. H. Hoogwater, Joris I. Erdmann, Andreas A. Schnitzbauer, Ernesto Sparrelid, Shishir K. Maithel, Cristina Dopazo, Abdul R. Hakeem, Francesca Ratti, Andrea Ruzzenente, Bas Groot Koerkamp

**Affiliations:** 1 Division of General and Hepato-Biliary Surgery, Department of Surgery, Dentistry, Gynaecology and Paediatrics, University of Verona, Verona, Italy; 2 Division of Hepato-Pancreato-Biliary Surgery and Transplantation, Department of Surgery, Erasmus MC Cancer Institute, Erasmus University, Rotterdam, The Netherlands; 3 Department of Surgery, Division of Hepato-Pancreato-Biliary Surgery and Liver Transplantation, University of Groningen, University Medical Center Groningen, Groningen, The Netherlands; 4 Department of Surgery, Cancer Center Amsterdam, Amsterdam UMC, University of Amsterdam, Amsterdam, The Netherlands; 5 Klinik für Allgemein-, Viszeral- und Transplantationschirurgie, Universitätsklinikum Frankfurt, Frankfurt, Germany; 6 Division of Surgery, Department of Clinical Science, Intervention and Technology (CLINTEC), Karolinska Institutet, Karolinska University Hospital, Stockholm, Sweden; 7 Division of Surgical Oncology, Winship Cancer Institute, Emory University, Atlanta, GA USA; 8 Department of HBP Surgery and Transplants, Vall d’Hebron Hospital Universitari, Vall d’Hebron Institut de Recerca (VHIR), Vall d’Hebron Barcelona Hospital Campus, Universitat Autónoma de Barcelona, Barcelona, Spain; 9 Division of Surgery, Department of Hepatobiliary and Liver Transplant Surgery, St James’s University Hospital, Leeds, UK; 10 Hepatobiliary Surgery Division, IRCCS San Raffaele Hospital, Milan, Italy

**Keywords:** Biliary tract cancer, Vascular resection, Vascular reconstruction, Perihilar cholangiocarcinoma, Oncological outcomes

## Abstract

**Background:**

Hepatectomy with associated vascular resection and reconstruction (VR) is an option to increase the number of patients with locally advanced perihilar cholangiocarcinoma (pCCA) eligible for radical-intent surgery.

**Objectives:**

This study aimed to assess the safety and oncological outcomes of VR in pCCA patients.

**Methods:**

Patients who underwent surgery for pCCA at 10 western centers were retrospectively reviewed and divided according to the performance of the VR. Primary outcomes were major morbidity, vascular morbidity, 90-day mortality, and overall survival (OS).

**Results:**

A total of 1054 patients were included, of whom 259 (24.6%) underwent VR. Of these 259 patients, 199 (76.8 %) underwent portal vein reconstruction (PVR) only and 60 (23.2%) underwent hepatic artery reconstruction (HAR) with or without PVR. VR patients were younger (66 vs. 68 years; *p* = 0.011) and more frequently had Bismuth type 4 tumors (31.3% vs. 22.9%; *p* = 0.008). They more frequently underwent portal vein embolization (32.0% vs. 17.6%; *p* < 0.001), biliary drainage (84.9% vs. 77.3%; *p* = 0.008), and extended hepatectomy (56.8% vs. 37.1%; *p* < 0.001), with longer operative times (539 vs. 479 min; *p* < 0.001) and higher blood loss (1300 vs. 700 mL; *p* < 0.001). Positive resection margins were observed more frequently (45.7% vs. 35.2%; *p* = 0.003). Major complications (51.4% vs. 41.0%; *p* = 0.004), vascular complications (19.7% vs. 3.3%; *p* < 0.001), and mortality (16.2% vs. 10.6%; *p* = 0.02) were higher in VR patients. Median OS was 28.0 months for patients without VR versus 22.8 months for patients with VR (*p* = 0.18).

**Conclusions:**

Liver resection and VR in patients with locally advanced pCCA are associated with increased major and vascular morbidity but offer similar survival as patients not undergoing VR; therefore, VR should be considered in selected patients.

**Supplementary Information:**

The online version contains supplementary material available at 10.1245/s10434-025-18137-4.

Perihilar cholangiocarcinoma (pCCA) is a rare and aggressive disease involving the hepatic hilum. Radical surgery, including major hepatectomy, hilar lymphadenectomy, and ipsilateral vascular and bile duct resection, is the treatment option that offers the best chance of long-term survival. Five-year overall survival (OS) after resection can be as high as 47%.^1–3^ Unfortunately, only a minority of pCCA patients are deemed resectable at diagnosis, mainly due to locally advanced disease.^4,5^

Involvement of the bilateral or main portal vein (PV), or bilateral or main hepatic artery (HA) precludes resection without vascular reconstruction (VR). Vascular resection with subsequent reconstruction of the PV and/or HA may allow for a complete resection in patients with locally advanced pCCA.^6^ Although VR has been increasingly performed in the last decades, previous studies were small and had conflicting results. Moreover, most patients underwent PV reconstruction (PVR), while HA reconstruction (HAR) was only performed in a minority of cases, increased surgical risks, and was generally considered to be oncologically futile. The main concern regarding VR is increased morbidity and mortality.^7–11^ Moreover, long-term oncological outcomes after resection are usually worse in patients with locally advanced cancer.^12,13^

The aim of this study was to compare surgical and oncological outcomes of VR between patients with and without VR, in a Western multicenter retrospective study.

## Methods

### Study Population

Patients undergoing surgical exploration at 25 European and American centers for proven or suspected pCCA, during any time span not preceding the year 2000, were included in a collaborative retrospective database of the Perihilar Cholangiocarcinoma Collaboration Group. The participating centers were required to provide complete information on VR and vascular-related complications. VR could have been preoperatively planned or decided intraoperatively for local invasion; patients requiring VR due to intraoperative mishap were not included. Ten of 25 centers were able to provide the additional data required for this study. Data were collected through a standardized, de-identified data file. Patients who underwent surgical exploration without resection as well as patients who underwent liver transplantation (LT) were not considered for this study. Moreover, only patients with pCCA at final surgical specimen examination were included; other cancers and dysplasia without cancer were excluded. Finally, patients undergoing bile duct resection without liver resection were excluded. The Institutional Medical Ethics Committee of the Erasmus MC waived the need for ethical approval and consent.

### Definitions

The work-up and management of patients, as well as postoperative management and follow-up differed across centers and during the inclusion period, according to the protocols of each institution. Tumors were classified according to the Bismuth–Corlette classification. Preoperative cholangitis was defined as fever and leucocytosis requiring biliary drainage or additional drainage. Liver resections were defined according to Brisbane terminology;^14^ perioperative transfusions were defined as transfusions of red blood cells occurring during surgery or within the first 48 h. Negative resection margins were defined as tumor-free margins in all the resection planes in the pathology report; tumors were staged according to the American Joint Committee on Cancer (AJCC) 8th edition TNM staging system.^15^

VR was defined as any resection of the right, left, or main PV or HA, followed by reconstruction; PVR was defined as the resection of a wedge portion or a segment of the right, left, or main PV, followed by reconstruction through direct suture/end-to-end anastomosis or graft interposition; and HAR was defined as a segmental resection of the right, left, or main HA, and reconstruction through end-to-end anastomosis, venous, or arterial graft interposition or use of rotated splenic artery. Patients undergoing both PVR and HAR were included in the HAR group. Resection of the middle HA (when present) without reconstruction, and resection of the HA without reconstruction in the presence of aberrant right or left arteries originating from the superior mesenteric or left gastric artery were not considered as VR. Reconstructions required for intraoperative vascular injuries rather than reconstruction for oncological reasons were not included in the VR group.

All complications registered during initial hospitalization or within 30 days after surgery were reported and classified according to the Clavien–Dindo classification.^16^ The definitions and grading for post-hepatectomy liver failure (PHLF), bile leak, and post-hepatectomy hemorrhage proposed by the International Study Group of Liver Surgery (ISGLS) were used. Only grades B and C were considered clinically relevant.^17–19^ Vascular complications, including thrombosis, bleeding, and stenosis were recorded for both PVR and HAR. Moreover, for HAR, liver infarction and pseudo-aneurysm were also recorded. OS was defined as the time between surgery and death or last follow-up.

### Statistical Analysis

Continuous variables were expressed as median and interquartile range (IQR), while categorical variables were expressed as numbers and percentages. The Mann–Whitney U test was used to compare continuous variables, and Fisher’s exact test or Pearson’s Chi-square test were used, where appropriate, for categorical variables. All tests were two-sided and a *p* value <0.05 was considered to indicate statistical significance. Multivariable analyses to identify independent prognostic factors for 90-day mortality and vascular complication were performed using logistic regression analysis. OS was analyzed using the Kaplan–Meier method and was compared using the log-rank test. Cox regression analysis was used to identify independent prognostic factors for OS. Survival analysis was performed without excluding patients who died within 90 days from surgery. The threshold for including factors from univariable analysis into multivariable analysis was *p* < 0.2. However, considering the main goals of this study, when performing Cox regression analysis for survival, VR, PVR, and HAR have been included by default, regardless of their *p* values on univariable analysis. All statistical analyses were performed, and all figures were created, using SPSS software version 28 (IBM Corporation, Armonk, NY, USA).

## Results

**Fig. 1:**
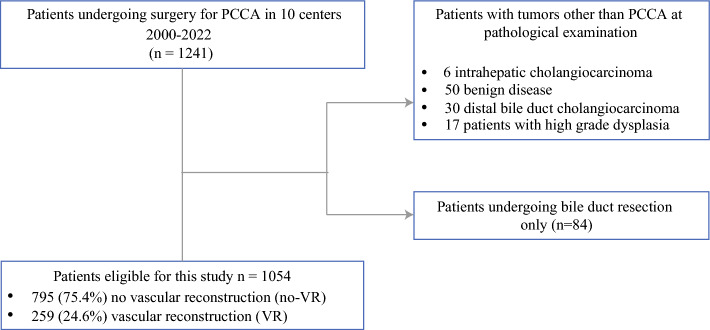
Selection of the study population among patients undergoing surgical exploration in the centers included in the analysis. *pCCA* perihilar cholangiocarcinoma

During the study period, 1241 patients underwent surgical exploration for pCCA in the 10 participating centers (Fig. 1). A total of 187 patients (15.1%) were excluded; 86 patients (6.9%) had a diagnosis other than pCCA at pathological examination, 17 had high-grade dysplasia without invasive cancer, and 84 patients underwent bile duct resection without partial hepatectomy. This resulted in a study population comprising 1054 patients who were divided into two groups: patients without VR (*n* = 795) and patients with VR (*n* = 259). VR patients were further subdivided into patients undergoing PVR (*n* = 199) and patients undergoing HAR with or without PVR (*n* = 60). Among the patients undergoing HAR, 42 had combined PVR and HAR, and 19 had HAR alone.

### Demographic, Operative, and Pathological Outcomes

Patients undergoing VR were younger than non-VR patients (66 [56–71] years vs. 68 [59–73] years; *p* = 0.011) and were more likely to have a higher bilirubin level, both at diagnosis and preoperatively (Table 1). Preoperative biliary drainage (84.9% vs. 77.3%; *p* = 0.008) and PVE (332.0% vs. 17.6%; *p* < 0.001) were performed more frequently in VR patients, who also experienced preoperative cholangitis more frequently.

**Table 1:** Demographic, operative, and pathological outcomes of patients undergoing VR compared with those who did not

Characteristics	No VR [*n* = 795]	VR [*n* = 259]	*p* value
Age, years	68 (59–73)	66 (56–71)	*0.011*^a^
Sex, male	490 (61.6)	164 (63.3)	0.66
BMI, kg/m^2^	25 (23–27)	25 (22–27)	0.26^a^
CA19-9 at diagnosis, U/mL	157 (46–619)	197 (46–593)	0.41^a^
Total bilirubin at diagnosis, umol/L	45 (12–159)	118 (26–200)	*<0.001*^a^
ASA score			
1–2	444 (66.8)	171 (73.1)	
3–4	221 (33.2)	63 (26.9)	0.09
PSC	20 (2.8)	12 (5.3)	0.09
Tumor diameter, cm	2.8 (2.0–3.7)	2.8 (2.0–3.8)	0.83
Bismuth classification			
Type 1, 2, 3	613 (77.1)	178 (68.7)	
Type 4	182 (22.9)	81 (31.3)	*0.008*
Preoperative biliary drainage	610 (77.3)	220 (84.9)	*0.008*
Preoperative cholangitis	158 (22.9)	83 (34.4)	*<0.001*
Preoperative PVE	140 (17.6)	83 (32.0)	*<0.001*
Preoperative total bilirubin, umol/L	14 (6–34)	17 (10–41)	*<0.001*^a^
Resection type			
S4/5 or central hepatectomy	50 (6.3)	4 (1.5)	
Left hemihepatectomy	274 (34.5)	55 (21.2)	
Right hemihepatectomy	176 (22.1)	53 (20.5)	
Left extended hepatectomy	85 (10.7)	29 (11.2)	
Right extended hemihepatectomy	210 (26.4)	118 (45.6)	*<0.001*^b^
Laterality, right side	426 (54.3)	173 (67.3)	*<0.001*
Extended hepatectomies	295 (37.1)	147 (56.8)	*<0.001*
S1 resection	497 (71.0)	203 (83.2)	*<0.001*
Combined pancreatectomy	14 (2.0)	8 (3.3)	0.32
Operative time, min	479 (360–580)	539 (439–616)	*<0.001*^a^
Estimated blood loss, mL	700 (500–1500)	1300 (700–2445)	*<0.001*^a^
Perioperative RBC transfusions	188 (26.0)	117 (49.4)	*<0.001*
Positive margin	280 (35.2)	118 (45.7)	*0.003*
AJCC staging, 7th edition, pT status			
pTis, pT1, pT2a/b	497 (64.5)	108 (42.0)	
pT3, pT4	273 (35.5)	149 (58.0)	*<0.001*
Positive lymph nodes	332 (42.3)	119 (46.5)	0.25
Distant metastases	40 (5.2)	16 (6.8)	0.33
Differentiation grading			
G1–2	561 (75.4)	175 (72.3)	
G3	183 (24.6)	67 (27.7)	0.35
Perineural invasion	559 (73.3)	222 (86.7)	*<0.001*

Continuous variables are expressed as median (IQR) and categorical variables are expressed as frequency (%)

Italicized *p* values indicate statistical significance

*VR* vascular resection, *BMI* body mass index, *CA19-9* carbohydrate antigen 19-9, *ASA* American Society of Anesthesiologists, *PVE* portal vein embolization, *RBC* red blood cells, *IQR* interquartile range, *AJCC* American Joint Committee on Cancer, *PSC* primary sclerosis cholangitis

Fisher’s exact test was used unless otherwise specified

^a^Mann–Whitney *U* test

^b^Pearson’s Chi-square test

Missing values (variables with more than 50 missing values are reported): BMI: 275 (24.1%); CA19-9 at presentation: 367 (32.2%); bilirubin at presentation: 219 (19.2%); tumor diameter: 276 (24.1%); preoperative total bilirubin: 70 (6.1%); estimated blood loss: 344 (30.2%); operative time: 216 (18.9%); ASA score: 172 (15.1%); PSC: 124 (10.9%); preoperative cholangitis 137 (12.0%); S1 resection: 121 (10.6%); pancreatoduodenectomy: 119 (10.5%); perioperative transfusion: 110 (9.7%); differentiation grading: 69 (6.1%)

VR patients more frequently underwent right-sided hepatectomies (67.3% vs. 54.3%; *p* < 0.001) and extended hepatectomies (56.8% vs. 37.1%; *p* < 0.001). Operative time was longer (539 vs. 479 min; *p* < 0.001), estimated blood loss was higher (1300 vs. 700 mL; *p* < 0.001), and perioperative transfusions were more frequent (49.4% vs. 26.0; *p* < 0.001) in VR patients. Finally, VR patients were more likely to have Bismuth type 4 tumors (31.3% vs. 22.9%; *p* = 0.008), a higher pT stage (pT3-4: 58.0% vs. 35.5%; *p* < 0.001), perineural invasion (86.7% vs. 73.3%; *p* < 0.001), and a positive margin (45.7% vs. 35.2%; *p* = 0.003). No differences were found in the proportion of patients with positive lymph nodes between VR and non-VR patients (46.5% vs. 42.3%; *p* = 0.25).

Demographic, operative, and pathological characteristics were also compared between PVR and HAR patients (electronic supplementary material [ESM] Table S1). The median carbohydrate antigen (CA) 19-9 level at diagnosis was higher for PVR patients (224 vs. 125; *p* = 0.015). PVR patients were more likely to undergo extended resections (67.3% vs. 21.7%; *p* < 0.001), with higher blood loss (1500 vs. 1000 mL; *p* = 0.013), while HAR patients had longer operative times (590 vs. 518 min; *p* = 0.004). Pathological reports of HAR patients more frequently showed pT3-4 stage tumors (76.7% vs. 52.3%; *p* < 0.001) and perineural invasion (94.9% vs. 84.3%; *p* = 0.047).

Most PVRs (84.9%) were segmental resections and reconstruction was mostly (96.5%) performed through end-to-end anastomosis. A graft was used in only 8 patients (3.5% of all PVRs) [Table 2]. End-to-end anastomosis was also the preferred reconstruction technique for HAR in 88.4% of patients.

**Table 2:** Characteristics of vascular resection and reconstruction patients

Characteristic	PVR [*n* = 199]	HAR [*n* = 60]
Type of PV resection		[*n* = 42]
Wedge	30 (15.1)	15 (35.7)
Segmental	169 (84.9)	27 (64.3)
Type of PV reconstruction		[*n* = 42]
End-to-end anastomosis	192 (96.5)	41 (93.6)
Graft interposition	7 (3.5)	1 (2.4)
Type of HA reconstruction	NA	
End-to-end anastomosis		54 (88.4)
Use of rotated splenic artery		2 (3.3)
Arterial graft interposition		3 (5.0)
Venous graft interposition		2 (3.3)

Data are expressed as frequencies (%)

*PVR* portal vein reconstruction, *HAR* hepatic artery reconstruction, *PV* portal vein, *HA* hepatic artery, *NA* not applicable

### Postoperative Outcomes

Patients undergoing VR had worse postoperative outcomes (Table 3). The incidence of 90-day mortality was 16.2% with VR and 10.6% without VR (*p* = 0.02), while the incidence of major complications was 51.4% with VR and 41.0% without VR (*p* = 0.004). PHLF grade B/C and infectious complications were more frequent in VR patients (22.4% vs. 13.0%, *p* < 0.001; and 24.6% vs. 18.1%, *p* = 0.03 respectively), but no differences were found in the occurrence of post-hepatectomy hemorrhage. Vascular complications occurred in 19.7% of patients after VR versus 3.3% of patients without VR (*p* ≤ 0.001). Both PV- and HA-related complications were more common in VR patients (14.3% vs. 1.4%, *p* < 0.001; and 5.8% vs. 2%, *p* = 0.005, respectively), and PV-related complications were more likely to be major complications in VR patients (9.6% vs. 0.5%, *p* < 0.001).

**Table 3:** Postoperative course of patients undergoing VR compared with those who did not

Characteristics	No VR [*n* = 795]	VR [*n* = 259]	*p* value
Length of hospital stay, days	14 (10–21)	17 (11–28)	*<0.001*^a^
30-day mortality	56 (7.0)	32 (12.4)	*0.01*
90-day mortality	84 (10.6)	42 (16.2)	*0.02*
Bleeding	9 (10.7)	8 (19.0)	
Liver failure	34 (40.5)	18 (42.9)	
Sepsis	28 (33.3)	14 (33.3)	
Other	13 (15.5)	2 (4.8)	0.24
Major complications (CD ≥3)	326 (41.0)	133 (51.4)	*0.004*
Liver failure, ISGLS grade B/C	103 (13.0)	58 (22.4)	*<0.001*
Bile leak, ISGLS grade B/C	159 (20.0)	48 (18.5)	0.65
Hemorrhage, ISGLS grade B/C	59 (7.4)	27 (10.4)	0.15
Intra-abdominal abscess	157 (19.8)	64 (24.7)	0.10
Infectious complication (CD ≥3)	142 (18.1)	63 (24.6)	*0.03*
Vascular complications	26 (3.3)	51 (19.7)	*<0.001*
PV-associated complications	11 (1.4)	37 (14.3)	*<0.001*
Thrombosis	5	24	
Bleeding	2	4	
Stenosis	4	10	
PV-associated complications (CD ≥3)	4 (0.5)	25 (9.6)	*<0.001*
Timing of PV complications			
Within 14 days from surgery	7 (63.6)	24 (64.9)	>0.99
HA-associated complications	16 (2.0)	15 (5.8)	*0.005*
Thrombosis	5	10	
Bleeding	7	3	
Liver infarction	5	3	
Stenosis	0	1	
Pseudoaneurysm	2	2	
HA-associated complications (CD ≥3)	14 (1.8)	10 (3.8)	0.06
Timing of HA complications			
Within 14 days from surgery	11 (73.3)	9 (60)	0.70

Continuous variables are expressed as median (IQR) and categorical variables are expressed as frequency (%)

Italicized *p* values indicate statistical significance

*VR* vascular resection, *ISGLS* International Study Group for Liver Surgery, *PV* portal vein, *HA* hepatic artery, *IQR* interquartile range, *CD* Clavien–Dindo

Fisher’s exact test was used unless otherwise specified

^a^Mann–Whitney U test

Missing values (variables with more than 50 missing values are reported): length of hospital stay: 96 (8.4%); adjuvant treatment: 220 (19.3%)

When comparing PVR and HAR patients, PVR patients were more likely to develop major complications (55.3% vs. 38.3%; *p* = 0.027) [ESM Table S2]. Only HA-related major complications were higher in HAR patients (16.7% vs. 2.5%; *p* ≤ 0.001).

Multivariable analyses were conducted for major (Clavien–Dindo grade 3 or higher) complications, vascular complications, and 90-day mortality (ESM Tables S3, S4, and S5, respectively); VR was independently associated with major complications (odds ratio [OR] 1.41, 95% confidence interval [CI] 1.03–1.93; *p* = 0.033) and vascular complications (OR 7.68, 95% CI 4.48–13.16; *p* < 0.001), but not for 90-day mortality. Other risk factors for major complications were preoperative cholangitis (OR 2.46, 95% CI 1.80–3.37; *p* < 0.001), with a tendency for significance for preoperative drainage (OR 1.42, 95% CI 0.98–2.06; *p* = 0.06) and extended resection (OR 1.32, 95% CI 0.98–1.93; *p* = 0.07). An American Society of Anesthesiologists (ASA) score of 3–4 (OR 1.93, 95% CI 1.13–3.30; *p* = 0.016) and preoperative cholangitis (OR 1.73, 95% CI 1.01–2.97; *p* = 0.045) were other independent prognostic factors for vascular complications. Other independent prognostic factors for 90-day mortality were age (OR 1.04, 95% CI 1.02–1.07; *p* = 0.001), male sex (OR 1.66, 95% CI 1.03–2.70; *p* = 0.036), preoperative cholangitis (OR 1.84, 95% CI 1.16–2.91; *p* = 0.009), right-sided resection (OR 1.69, 95% CI 1.02–2.81; *p* = 0.04) and extended resections (OR 1.94, 95% CI 1.21–3.01; *p* = 0.006).

### Survival Analysis

**Fig. 2:**
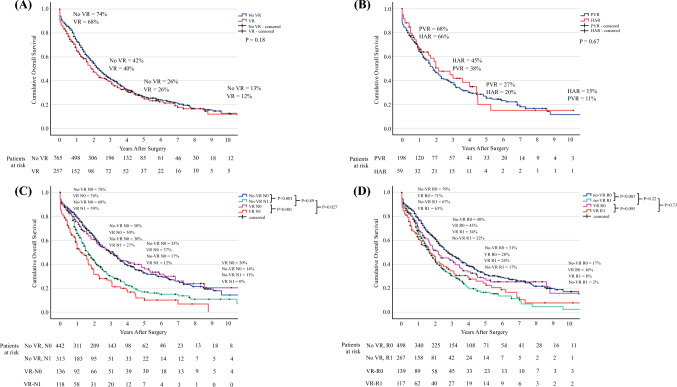
Kaplan–Meier curves for overall survival. **a** VR patients versus non-VR patients; **b** patients undergoing VR, comparing PVR and HAR; **c** VR patients versus non-VR patients with or without node-positive disease; and **d** VR patients versus non-VR patients with or without margin-positive disease. *VR* vascular resection, *PVR* portal vein reconstruction, *HAR* hepatic artery reconstruction

The median OS was 22.8 months (18.6–27.1) for VR patients and 28.0 months (24.7–31.3) for non-VR patients, with no differences being found (*p* = 0.18), or when comparing HAR and PVR patients (median OS: 26.0 [11.1–40.9] vs. 22.0 [167–27.3] months; *p* = 0.67). Among patients who underwent VR, OS was 26.0 months (16.3–35.7) in the case of R0 resection and 20.0 months (13.7–26.3) in the case of R1 resection (*p* = 0.095) [Fig. 2]. Advanced age, higher body mass index (BMI), positive margin, pT3-4 stage, positive lymph nodes, pM1, poor (G3) tumor differentiation, and perineural invasion were independent poor prognostic factors for worse OS. VR, PVR, and HAR were not associated with worse survival (Table 4).

**Table 4:** Cox regression analysis for overall survival in the study population

Variable	Univariable		Multivariable	
	Hazard ratio	*p* value	Hazard ratio	*p* value
Age, years	1.01 (1.00–1.02)	0.02	1.02 (1.01–1.03)	*0.035*
Sex, male	1.25 (1.06–1.47)	0.008	1.21 (0.90–1.63)	0.21
BMI	1.02 (0.99–1.04)	0.06	1.03 (0.99–1.07)	0.077
CA19-9 >37 U/mL	1.32 (1.04–1.68)	0.02	1.25 (0.86–1.80)	0.23
Bismuth type 4	1.31 (1.11–1.56)	0.002	1.20 (0.86–1.68)	0.29
Pancreatoduodenectomy	1.01 (0.60–16.8)	0.98	–	
Positive margin	1.52 (1.29–1.78)	<0.001	1.30 (1.00–1.68)	*0.05*
pT3/pT4 tumors	1.37 (1.14–1.59)	<0.001	1.50 (1.12–1.99)	*0.006*
Positive lymph nodes	1.70 (1.45–2.00)	<0.001	1.50 (1.14–2.04)	*0.004*
Distant metastases (pM1)	1.82 (1.36–2.42)	<0.001	2.08 (1.28–3.40)	*0.003*
Poor (G3) differentiation	1.78 (1.48–2.13)	<0.001	1.46 (1.08–1.99)	*0.015*
Perineural invasion	1.40 (1.13–1.72)	0.001	2.13 (1.30–3.49)	*0.003*
Vascular Reconstruction	1.13 (0.95–1.34)	0.18	0.62 (0.15–2.60)	0.51
PVR	1.14 (0.95–1.38)	0.17	1.08 (0.80–1.48)	0.63
HAR	1.07 (0.76–1.52)	0.70	1.24 (0.63–2.46)	0.54

Only variables with a *p* value <0.20 at univariate analysis were included in the multivariate analysis

Italicized *p* values indicate statistical significance

*BMI* body mass index, *CA19-9* carbohydrate antigen 19-9, *PVR* portal vein reconstruction, *HAR* hepatic artery reconstruction

## Discussion

This multicenter study compared the results, after resection of pCCA, of more than 1000 patients with and without VR. Approximately one in four patients underwent VR, of whom approximately one in four had an HAR. VR patients had a higher risk of major complications (51.4% vs. 41.0%; *p* = 0.004), vascular complications (3.3% vs. 19.7%; *p* < 0.001), and mortality (16.2% vs. 10.6%; *p* = 0.02). However, the median OS was 28.0 months for patients without VR versus 22.8 months for patients with VR (*p* = 0.18).

In a recent paper from the Nagoya University, Mizuno et al. compared outcomes of pCCA patients with and without VR. In their impressive monocentric retrospective cohort of 787 pCCA patients who underwent surgery (303 with VR), the incidence of major complications was 48% versus 50% (*p* = 0.715) in patients with VR and without VR; however, 90-day mortality was 3.6% for patients with VR and 1.2% (*p* = 0.04) for patients without VR.^8^ While the incidence of major complications was similar, 90-day mortality was much lower. This very low mortality rate could be explained by differences in patient population, perioperative care, and/or intraoperative technique.^20^ In their paper published in 2021, Mueller et al. extracted benchmark values for pCCA surgery from 1829 consecutive patients in a 5-year period from 30 centers worldwide.^1^ In the present study, VR patients had better outcomes than the published benchmark outcomes for major complications [≤ 70%, PHLF (≤ 22.5%), and bile leak (≤ 47%)]. Postoperative 90-day mortality in VR patients was 16% and exceeded the benchmark of 13%. However, for patients undergoing PVR, the 90-day mortality was 26.6% in the benchmark cohort.

Major complications, vascular complications, and 90-day mortality were more frequent in VR patients, and VR was independently associated with major complications (OR 1.41; *p* = 0.033) and vascular complications (OR 7.68; *p* < 0.001), but not 90-day mortality. Other authors reported that VR was associated with more vascular complications. Lemaire et al. reported an 8% incidence of PV thrombosis in their cohort of 86 patients undergoing surgery for pCCA; all thromboses occurred in patients undergoing VR.^21^ However, other factors have an impact on morbidity and mortality for these patients. Remarkably, preoperative cholangitis was associated with both major and vascular complications as well as 90-day mortality; extended resections and right-sided resections were associated with 90-day mortality alone. These results that are in agreement with the available literature.^22–29^

Finally, with 60 patients included, this multicenter study is the largest Western series of HAR.^7^ The most frequently associated liver resection performed in these patients was left hepatectomy, a result similar to other reports, which is due to the anatomy of the hilum, since the left HA runs far from the confluence while the right HA typically lies close to the biliary confluence. Therefore, left-sided pCCA with involvement of the right HA may be frequent.^7,8^ In our case series, HAR had comparable results as PVR alone, while in other series, outcomes after HAR are dismal. In one of the few Western series, Schimizzi et al. reported a 67% rate of severe major complications for HAR in 12 patients,^30^ while other high-volume reports from Eastern countries show severe morbidity incidence rates ranging between 19 and 66%.^8,31,32^

Among the most important results of this paper is the fact that VR patients had similar OS compared with non-VR patients: median survival and 5-year OS were 22.8 months (18.6–27.1) and 26%, respectively, compared with 28.0 months (24.7–31.3) and 26%, respectively, for non-VR (*p* = 0.18). VR was also not a poor prognostic factor after adjusting for tumor extension (pT stage), nodal involvement, poor differentiation, perineural invasion, positive margins, and distant metastases, nor were HAR or PVR considered separately. Similar results were reported by She et al.^33^ Patients who underwent VR usually have higher tumor stage with a higher incidence of positive nodal status. In their paper, Mizuno et al. reported a significantly higher incidence of nodal metastases in VR patients than in non-VR patients (62–64% vs. 37%, *p* < 0.001);^8^ however, in our cohort, we did not observe significant differences in nodal stage. We confirmed the prognostic role of nodal status in both VR and non-VR. Figure 2d depicts survival stratified for radicality: R1 patients have significantly different curves in the case of no VR, while the curves for VR patients separate and show a trend to significance (*p* = 0.095). These findings may be related to a more difficult margin evaluation in VR patients: margin assessment differs among centers, and, in particular, there is no agreement as to how to interpret the radial margin, i.e. the extension into the peritoneum of the hepatoduodenal ligament, as opposed to the ductal margin, i.e. the extension distally and proximally along the bile ducts. It has been proven that true R0 patients, i.e. patients with ductal and radial negative margins, are those with the best survival, but the radial margin is not always expressed in the pathological report.^34–36^ Patients requiring VR have a vascular invasion in the hepatoduodenal ligament, therefore it is highly probable that these confusing results are due to unreported radial margin involvement, explaining the lack of statistical significance.

The impact of VR for locally advanced pCCA can only be definitively investigated with a randomized controlled trial (RCT), which would be challenging and is unlikely to accrue sufficient patients. Nevertheless, the median OS (22.8 months [18.6–27.1]) and 5-year OS (26%) of the VR cohort presented in this study was favorable, considering that 5-year survival is rare without resection. Mizuno et al. reported 3- and 5-year OS rates of 4.0% and 2.7%, respectively, for pCCA patients not undergoing surgery, which was significantly worse than their VR patient cohort (43.6% and 27.0, respectively).^8^ Ruys et al. reported that 7.0% of pCCA patients without a resection survived at least 5 years from diagnosis;^37^ a ‘real world’ comparison could be performed with unresectable patients undergoing chemotherapy, such as those included in the TOPAZ trial.^38^ In that study, the 198 patients undergoing gemcitabine/cisplatin plus durvalumab chemotherapy showed a median OS of 12.8 and 2-year OS of 24.9%, results that are still worse than the 27% and 30% 3-year OS of VR and non-VR patients with positive lymph nodes. This potential improvement in OS should be weighed against the increased risk of major and vascular complications for VR of pCCA in the present study, and the negative impact that positive lymph nodes have.

An alternative option for patients with locally advanced pCCA is orthotropic LT with or without intensive neoadjuvant chemoradiation.^39,40^ A recent benchmarking paper was published by Breuer et al., analyzing 134 patients undergoing LT for pCCA in Europe and North America in a 5-year period.^39^ The benchmark 5-year OS after LT in patients was ≥ 60% compared with the more unfavorable 5-year OS of 26% in the present study. Moreover, in the present study, the 90-day mortality benchmark value was ≤ 5.2% after LT versus 16.6% after VR. Unfortunately, the selection criteria for LT are strict, and LT for pCCA is not an option in all countries. As an example, Vugts et al. reported that only about 5% of all 732 patients referred for pCCA to two tertiary Dutch centers would have fulfilled the Mayo criteria for LT.^41^ Moreover, as reported by Croome et al., the survival results of the two techniques are more influenced by tumor biology (i.e. nodal status) than the technique implemented (LT vs. resection).^42^ Therefore, LT should be reserved for selected patients, while resectable de novo pCCA should be resected; however, the choice to offer surgery (either LR or resection) to a patient should be subordinated to a stratification based on well-known prognostic factors, such as nodal status. That said, no RCTs were available after the premature termination, due to failure of recruitment, of the TRANSPHIL trial (NCT02232932), while the first results for the LITHALICA trial (NCT06125769) are awaited not before the year 2028. Further investigations are needed to compare the results of VR in liver resection versus LT for pCCA patients.^43^

Some limitations of this study must be reported. First, this was a multicenter study, therefore biases regarding patient selection are unavoidable. Furthermore, the number of VRs performed is likely an overestimation of the real volumes of VR in the Western world, given the nature of our collaboration, which includes mostly high-volume centers. Second, preoperative and postoperative management protocols varied among centers. Third, the study involved patients enrolled over a long period of time, in which protocols of adjuvant chemotherapy have been developed and applied, changing the indications to surgery of patients with locally advanced pCCA.^44,45^ Finally, the analysis focused on patients undergoing resection, and a cohort of non-resected patients coming from the same centers was not available for comparison of the survival results.

## Conclusion

Liver resection and VR in patients with locally advanced pCCA is associated with increased major and vascular morbidity but offers similar survival as patients not undergoing VR; therefore, VR should be considered in selected patients.

## Supplementary Information

Below is the link to the electronic supplementary material.

Supplementary file1 (DOCX 35 kb)

## Data Availability

Data were obtained from the Perihilar Cholangiocarcinoma Collaboration Group. Data are available from the corresponding author upon reasonable request.
