# Supplementary material for: Operative and Oncological Outcomes of Vascular Resection and Reconstruction for Perihilar Cholangiocarcinoma
Source: Ann Surg Oncol. 2025 Sep 13;32(13):9597–607. doi: 10.1245/s10434-025-18137-4 (PMC12589212; doi:10.1245/s10434-025-18137-4)
Supplement: Supplementary file 1 — Supplementary file1 (DOCX 35 kb) [file 10434_2025_18137_MOESM1_ESM.docx]

SUPPLEMENTARY MATERIAL

Table S1: Demographic, operative and pathological outcomes of patients undergoing VR, divided in PVR and HAR patients.

| **Characteristics** | **PVR (n=199)** | **HAR (n=60)** | **P-value** |
| --- | --- | --- | --- |
| **Age, years** | 65 (56-71) | 66 (59-73) | 0.25*** |
| **Sex, male** | 123 (61.8%) | 41 (68.3%) | 0.45 |
| **BMI, kg/m^2^** | 25 (22-27) | 26 (22-28) | 0.14*** |
| **Ca19-9 at diagnosis, U/mL** | 224 (62-614) | 125 (23-397) | *0.015** |
| **Total bilirubine at diagnosis, umol/L** | 129 (36-207) | 86 (20-161) | 0.09*** |
| **ASA score**  **ASA 1-2**  **ASA 3-4** | 136 (75.1%)  45 (24.9%) | 35 (66.0%)  18 (34.0%) | 0.22 |
| **Primary sclerosis cholangitis** | 11 (6.5%) | 1 (1.7%) | 0.19 |
| **Tumor diameter at imaging, cm** | 2.7 (2.0-3.8) | 2.9 (2.0-3.5) | 0.91*** |
| **Bismuth classification**  **Type 1-2-3**  **Type 4** | 141 (70.9%)  58 (29.1%) | 37 (61.7%)  23 (38.3%) | 0.20 |
| **Preoperative biliary drainage** | 169 (84.9%) | 51 (85.0%) | >0.99 |
| **Preoperative cholangitis** | 60 (32.8%) | 23 (39.7%) | 0.35 |
| **Preoperative PVE** | 70 (35.2%) | 13 (21.7%) | 0.06 |
| **Preoperative total bilirubine, umol/L** | 17 (10-41) | 20 (9-32) | 0.96*** |
| **Resection type**  **S4/5 or central hepatectomy**  **Left hemihepatectomy**  **Right hemihepatectomy**  **Left extended hepatectomy**  **Right extended hemihepatectomy** | 1 (0.5%)  30 (15.1%)  34 (17.1%)  22 (11.1%)  112 (56.3%) | 3 (5.0%)  25 (41.7%)  19 (31.7%)  7 (11.6%)  6 (10.0%) | *<0.001*** |
| **Extended Hepatectomies** | 134 (67.3%) | 13 (21.7%) | *<0.001* |
| **S1 resection** | 150 (80.6%) | 53 (91.4%) | 0.07 |
| **Combined pancreatectomy** | 8 (4.3%) | 0 (0%) | 0.20 |
| **Operative time, min** | 518 (430-600) | 590 (539-629) | *0.004** |
| **Estimated blood losses, mL** | 1500 (800-2500) | 1000 (600-1500) | *0.013** |
| **Perioperative RBC transfusions** | 84 (47.2%) | 33 (55.9%) | 0.29 |
| **Positive margin** | 89 (44.7%) | 29 (49.2%) | 0.56 |
| **AJCC staging, 7^th^ ed, pT status**  **pTis, pT1, pT2a/b**  **pT3, pT4** | 94 (47.7%)  103 (52.3%) | 14 (23.3%)  46 (76.7%) | *<0.001* |
| **Positive lymph nodes** | 87 (44.2%) | 32 (54.2%) | 0.18 |
| **Distant metastases** | 13 (7.4%) | 3 (5.1%) | 0.77 |
| **Differentiation grading**  **G1-2**  **G3** | 137 (74.5%)  47 (25.5%) | 38 (65.5%)  20 (34.5%) | 0.24 |
| **Perineural invasion** | 166 (84.3%) | 56 (94.9%) | *0.047* |
| **Overall Survival, months** | 22.0 (16.7-27.3) | 26.0 (11.1-40.9) | 0.67§ |

Continuous variables are expressed as median and interquartile range (IQR), cathegorical variables are expressed as frequencies and percentages. values in italics are statistically significant. Abbreviations: VR: Vascular reconstruction; PVR: Portal vein reconstruction; HAR: hepatic artery reconstruction; PVE: portal vein embolization. RBC: red blood cells.

Fisher’s exact test was used when not oherwise specified.

* Mann-Whitney U test

** Pearson’s χ^2^ Test

§ Log rank test

Table S2: Postoperative course of patients undergoing VR, divided in PVR vs HAR patients.

| **Characteristics** | **PVR (n=199)** | **HAR (n=60)** | **PVR vs HAR** |
| --- | --- | --- | --- |
| **Length of hospital stay, days** | 18 (11-29) | 16 (11-28) | 0.57*** |
| **30-days mortality** | 27 (13.6%) | 5 (8.3%) | 0.37 |
| **90-days mortality** | 33 (16.6%) | 9 (15.0%) | 0.84 |
| **Major complications (C-D≥3)** | 110 (55.3%) | 23 (38.3%) | *0.027* |
| **Liver Failure, ISGLS grade B/C** | 46 (23.1%) | 12 (20.0%) | 0.72 |
| **Bile leak, ISGLS grade B/C** | 38 (19.1%) | 10 (16.7%) | 0.85 |
| **Hemorrage, ISGLS grade B/C** | 23 (11.6%) | 4 (6.7%) | 0.34 |
| **Intra-abdominal abscess** | 50 (25.1%) | 14 (23.3%) | 0.86 |
| **Infectious complication (C-D≥3)** | 53 (27.0%) | 10 (16.7%) | 0.12 |
| **Vascular complications** | 37 (18.6%) | 14 (23.3%) | 0.46 |
| **PV associated complications**  **Thrombosis**  **Bleeding**  **Stenosis**  **PV associated complications (C-D≥3)** | 33 (16.6%)  22  4  8  22 (11.1%) | 4 (6.7%)  2  0  2  3 (5.0%) | 0.06  0.21 |
| **PV compications timing**  **Within 14 days from surgery** | 22 (66.7%) | 2 (50.0%) | 0.60 |
| **HA associated compications**  **Thrombosis**  **Bleeding**  **Liver infarction**  **Stenosis**  **Pseudoaneurysm**  **HA associated complications (C-D≥3)** | 5 (2.5%)  4  1  1  0  0  4 (2.0%) | 10 (16.7%)  6  2  2  1  2  6 (10.0%) | *<0.001*  *0.012* |
| **HA compications timing**  **Within 14 days from surgery** | 4 (80.0%) | 4 (44.4%) | 0.29 |

Continuous variables are expressed as median and interquartile range (IQR), cathegorical variables are expressed as frequencies and percentages. values in italics are statistically significant. Abbreviations: VR: Vascular reconstruction; PVR: Portal vein reconstruction; HAR: hepatic artery reconstruction; C-D: Clavien-Dindo classification; ISGLS: International Study Group for Liver Surgery; Fisher’s exact test was used when not oherwise specified.

* Pearson’s Mann Whitney U test

Table S3: Uni and Multivariable logistic regression analysis for major complications in the study population.

| **Variable** | **Univariable** | | **Multivariable** | |
| --- | --- | --- | --- | --- |
|  | **Odds Ratio** | **P value** | **Odds Ratio** | **P value** |
| **Age, years** | 0.99 (0.98-1.01) | 0.43 | - | - |
| **Sex, male** | 1.18 (0.92-1.52) | 0.19 |  |  |
| **BMI** | 1.00 (0.97-1.04) | 0.83 | - | - |
| **ASA 3-4** | 1.05 (0.79-1.40) | 0.72 | - | - |
| **Preoperative drainage** | 1.69 (1.24-2.31) | 0.001 | 1.42 (0.98-2.06) | 0.06 |
| **Preoperative PVE** | 1.41 (1.05-1.89) | 0.024 | 1.18 (0.82-1.70) | 0.38 |
| **Preoperative cholangitis** | 2.80 (2.07-3.79) | <0.001 | 2.54 (1.85-3.49) | *<0.001* |
| **Bismuth, type 4** | 1.07 (0.81-1.42) | 0.62 | - | - |
| **Tumour diameter** | 0.95 (0.82-1.04) | 0.27 | - | - |
| **Vascular Reconstruction** | 1.52 (1.15-2.01) | 0.004 | 1.41 (1.03-1.93) | *0.033* |
| **Extended resection** | 1.57 (1.23-2.01) | <0.001 | 1.32 (0.98-1.93) | 0.07 |
| **Right sided resection** | 1.59 (1.24-2.04) | <0.001 | 1.28 (0.96-1.72) | 0.10 |
| **Pancreatoduodenectomy** | 2.48 (1.03-5.98) | 0.042 | 1.93 (0.77-4.84) | 0.16 |

Table S4: Uni and Multivariable logistic regression analysis for vascular complications in the study population.

| **Variable** | **Univariable** | | **Multivariable** | |
| --- | --- | --- | --- | --- |
|  | **Odds Ratio** | **P value** | **Odds Ratio** | **P value** |
| **Age, years** | 0.99 (0.97-1.02) | 0.61 | - |  |
| **Sex, male** | 1.30 (0.79-2.12) | 0.30 | - |  |
| **BMI** | 1.03 (0.98-1.09) | 0.22 | - |  |
| **ASA 3-4** | 1.49 (0.90-2.46) | 0.12 | 1.93 (1.13-3.30) | *0.016* |
| **Preoperative drainage** | 2.07 (1.02-4.22) | 0.045 | 1.74 (0.74-4.09) | 0.20 |
| **Preoperative PVE** | 0.89 (0.50-1.60) | 0.71 | - |  |
| **Preoperative cholangitis** | 1.90 (1.14-3.15) | 0.013 | 1.73 (1.01-2.97) | *0.045* |
| **Bismuth, type 4** | 1.40 (0.84-2.31) | 0.19 | 0.95 (0.52-1.72) | 0.86 |
| **Tumour diameter** | 1.03 (0.87-1.23) | 0.70 | - |  |
| **Vascular Reconstruction** | 7.52 (4.41-11.91) | <0.001 | 7.68 (4.48-13.16) | *<0.001* |
| **Extended resection** | 1.55 (0.97-2.46) | 0.07 | 0.88 (0.52-1.49) | 0.63 |
| **Right sided resection** | 1.16 (0.71-1.88) | 0.55 | - | - |
| **Pancreatoduodenectomy** | 0.58 (0.08-4.38) | 0.60 | - |  |

Only variables with a p value <0.20 at univariate analysis were included in multivariate analysis. P values in italics identify statistical significance.

Table S5: Uni and Multivariable logistic regression analysis for 90 Days Mortality in the study population.

| **Variable** | **Univariable** | | **Multivariable** | |
| --- | --- | --- | --- | --- |
|  | **Odds Ratio** | **P value** | **Odds Ratio** | **P value** |
| **Age, years** | 1.03 (1.01-1.06) | <0.001 | 1.04 (1.02-1.07) | *0.001* |
| **Sex, male** | 1.48 (0.99-2.21) | 0.06 | 1.66 (1.03-2.70) | *0.036* |
| **BMI** | 1.02 (0.98-1.07) | 0.32 | - |  |
| **ASA 3-4** | 1.52 (0.99-2.32) | 0.06 | 1.35 (0.84-2.15) | 0.20 |
| **Preoperative drainage** | 1.00(0.63-1.58) | >0.99 | - |  |
| **Preoperative PVE** | 1.31 (0.85-2.02) | 0.21 | - |  |
| **Preoperative cholangitis** | 1.74 (1.13-2.69) | 0.012 | 1.84 (1.16-2.91) | *0.009* |
| **Bismuth, type 4** | 1.53 (1.03-2.29) | 0.037 | 1.39 (0.85-2.28) | 0.19 |
| **Tumour diameter** | 1.04 (0.91-1.21) | 0.51 | - |  |
| **Vascular Reconstruction** | 1.64 (1.10-2.45) | 0.016 | 1.47 (0.92-2.36) | 0.11 |
| **Extended resection** | 1.89 (1.28-2.71) | 0.001 | 1.94 (1.21-3.01) | *0.006* |
| **Right sided resection** | 2.19 (1.42-3.30) | <0.001 | 1.69 (1.02-2.81) | *0.04* |
| **Pancreatoduodenectomy** | 0.81 (0.18-3.50) | 0.77 | - |  |

Only variables with a p value <0.20 at univariate analysis were included in multivariate analysis. P values in italics identify statistical significance.
